# A rapid seamless method for gene knockout in *Pseudomonas aeruginosa*

**DOI:** 10.1186/s12866-017-1112-5

**Published:** 2017-09-19

**Authors:** Weiliang Huang, Angela Wilks

**Affiliations:** Department of Pharmaceutical Sciences, School of Pharmacy, University of Maryland, Baltimore, MD USA

**Keywords:** *Pseudomonas Aeruginosa*, Genetic knockout, Gibson assembly

## Abstract

**Background:**

*Pseudomonas aeruginosa* is a model organism for the study of quorum sensing, biofilm formation, and also leading cause of nosocomial infections in immune compromised patients. As such *P. aeruginosa* is one of the most well studied organisms in terms of its genetics. However, the construction of gene deletions and replacements in *Pseudomonas aeruginosa* is relatively time-consuming, requiring multiple steps including suicide vector construction, conjugation, inactivation with insertion of antibiotic resistance cassettes and allelic exchange. Even employing Gateway recombineering techniques with direct transformation requires a minimum two weeks.

**﻿﻿Methods:**

We have developed a rapid streamlined method to create clean deletion mutants in *P. aeruginosa* through direct transformation, eliminating the need for the creation of Gateway-compatible suicide vectors. In this method, upstream and downstream sequences of the gene/locus to be deleted are amplified by polymerase chain reaction (PCR) and seamlessly fused with the linearized pEX18Tc *sacB* suicide plasmid by Gibson assembly. The resulting deletion plasmid is transformed into *P. aeruginosa* by an electroporation method optimized in this study. The plasmid is then integrated into the chromosome by homologous recombination, and deletion mutants are identified via *sacB* mediated sucrose counter-selection.

**﻿Results:**

T﻿he current method was employed to generate clean gene deletions of the heme assimilation system anti-σ factor, *hasS* and the virulence regulator involving ECF system anti-σ and σ factors *vreA* and *vreI*, respectively. The process from plasmid construction to confirmation by DNA sequencing of the gene deletion was completed in one week. Furthermore, the utility of the method is highlighted in the construction of the *vreA* and *vreI* deletions, where the start codon of vreA and the stop codon of vreI overlap. Utilizing Gibson assembly deletion mutants were constructed with single base pair precision to generate the respective *vreA* and *vreI* deletions, while maintaining the start and stop codon of the respective genes. Overall, this method allows for rapid construction of gene deletions in *P. aeruginosa *with base pair precision.

**Conclusion:**

This method from the construction of the suicide vector to sequence confirmation of the unmarked gene deletion can be performed in one week, without the requirement for expensive proprietary reagents or instruments. The precision of Gibson assembly and the fact the accuracy in generating the desirable construct is 95%, makes this a viable and attractive alternative to previous methods.

**Electronic supplementary material:**

The online version of this article (10.1186/s12866-017-1112-5) contains supplementary material, which is available to authorized users.

## Background

*Pseudomonas aeruginosa* is a common cause of hospital-acquired infections including respirator-associated pneumonias, bacteremias, and urinary tract and surgical site infections. *P. aeruginosa* accounts for 51,000 opportunistic infections/year with approximately 13% being multi-drug resistant [1, 2]. The *P. aeruginosa* genome is one of the largest bacterial genomes at approximately 6 million base pairs with about 65% guanine + cytosine content [3]. The adaptability of *P. aeruginosa* is reflected in the fact that the genome encodes 5500 open reading frames with a significant proportion of the genome dedicated to regulatory genes and genes associated with the breakdown, transport and efflux of organic compounds. The large and complex genome of *P. aeruginosa* provides unique challenges in terms of developing genetic tools by which to study the biological function of specific genes.

The generation of specific deletion mutants in *P. aeruginosa* has relied on allelic exchange by homologous recombination that requires 3–4 weeks [4, 5]. Hmelo et al., recently published an in-depth two-step allelic exchange protocol that can be applied to gene knockouts and knock-ins, as well as single-nucleotide insertions and deletions or substitutions [6]. The general method of two step allelic exchange requires cloning of the upstream and downstream sequences of a locus of interest into a suicide vector such as the pEX18 plasmids pioneered by the Schweizer laboratory [7]. Secondly, the deletion or mutant vector is transformed into a conjugative *E. coli* strain such as SM10. The mutant allele is then transferred into *P. aeruginosa* by biparental mating. The vector containing the deletion allele is integrated into the *P. aeruginosa* chromosome by in vivo homologous recombination. The resulting merodiploids are selected against the antibiotic resistance marker encoded within the integrated plasmid backbone. The isolated merodiploids undergo a second homologous recombination, which is counter-selected through the suicide marker *sacB* to isolate colonies that have lost the plasmid backbone. The completion of two-step allelic exchange takes upward of two weeks to complete. The use of Gateway technology to clone the mutant allele into a donor allelic exchange vector, which is then transformed into *P. aeruginosa* by electroporation, still requires at least two weeks [8].

Herein we describe an optimized and rapid method to create unmarked deletion mutants of *P. aeruginosa* in as few as seven days with total hands-on time of less than half a day*.* Briefly, allelic exchange constructs for gene deletion are generated by Gibson assembly [9] and introduced into *P. aeruginosa* using an optimized electroporation protocol. We have successfully created clean deletion mutants in the virulence regulator involving ECF system (*vre*, also known as PUMA3) and the heme assimilation system (*has*) of *P. aeruginosa* using this method. Herein we describe the generation of a clean deletion mutant of the anti- σ factor receptor *hasS*, the signaling receptor *vreA* and σ factor *vreI*, respectively. We expect this method can be readily adapted to create insertion and substitution mutants in other genes and for use in related organisms.

## Methods

### Media

*E. coli* DH5α (New England BioLabs) was maintained on lysogeny broth (LB) agar medium (10 g/L tryptone, 5 g/L yeast extract, 5 g/L NaCl, 15 g/L agar). *P. aeruginosa* PAO1 (obtained from Professor Michael Vasil, University of Colorado) [10] was maintained on brain-heart infusion (BHI) agar medium (5 g/L beef heart infusion, 12.5 g/L calf brains infusion, 2.5 g/L disodium hydrogen phosphate, 2 g/L glucose, 10 g/L peptone, 5 g/L sodium chloride, 15 g/L agar). For plasmid preparation, *E. coli* transformants were cultured in terrific broth (TB) (12 g/L peptone, 24 g/L yeast Extract, 9.4 g/L dipotassium hydrogen phosphate, 2.2 g/L potassium dihydrogen phosphate, 4 ml/L glycerol). Tryptone yeast extract and sucrose (TYS10) (10 g/L tryptone, 5 g/L yeast extract, 10% (*W*/*V*) filtered sucrose, 15 g/L agar) plates were used in the selection of the second homologous recombination event. Tetracycline at 10 μg/ml and 100 μg/ml were used to select *E. coli* transformants and *P. aeruginosa* merodiploids, respectively.

### Amplification of the upstream and downstream sequences of the locus of interest

**Fig. 1:**
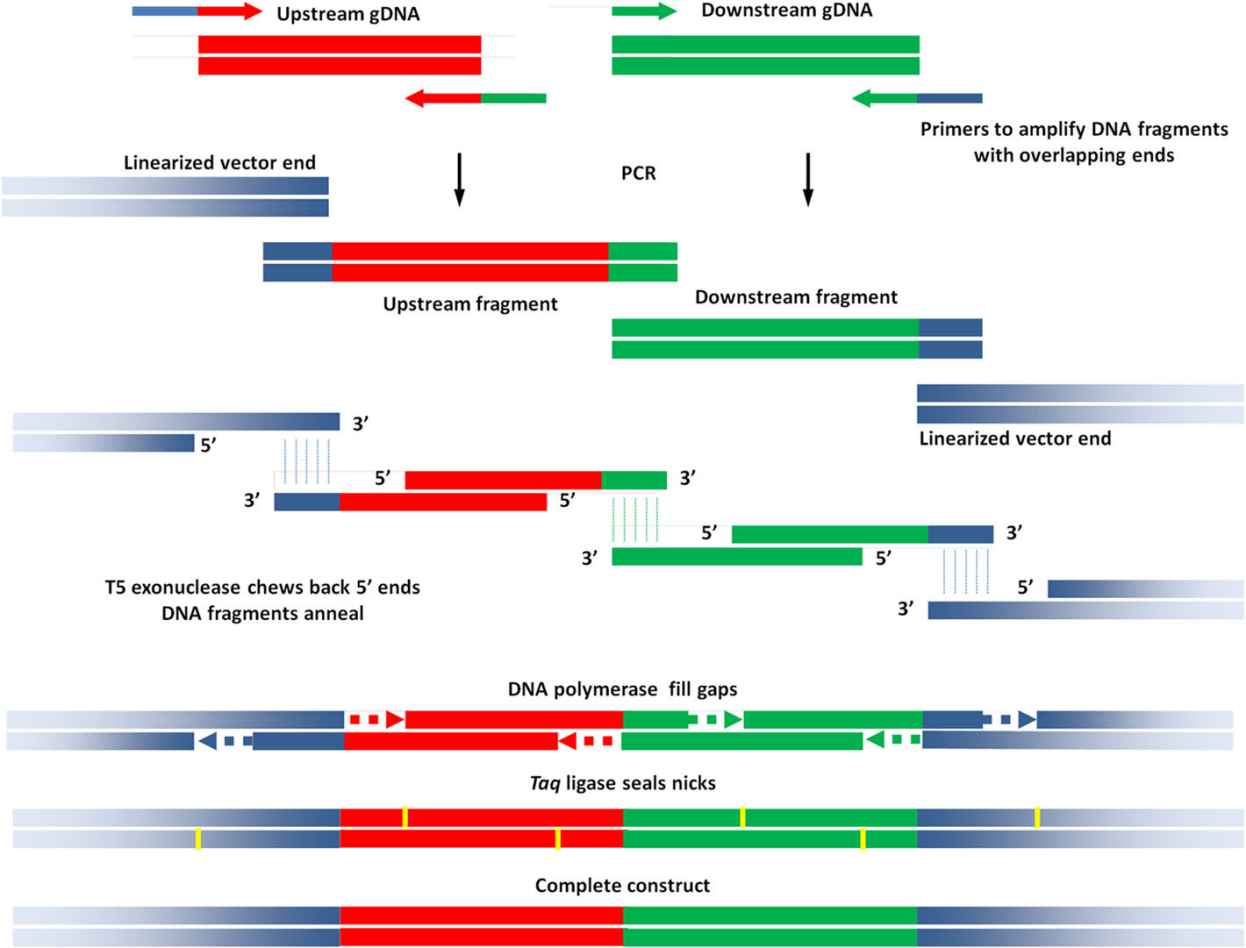
Construction of deletion construct via Gibson assembly. DNA fragment ends of the same color represent identical sequences, which facilitate DNA fusion. gDNA, genomic DNA

Sequence information was obtained from the *Pseudomonas* Genome Database (http://www.pseudomonas.com/). Partial overlapping flanking primers were designed to amplify the upstream and the downstream 500 bp DNA sequences of *hasS, vreI and vreA* using NEBuilder (http://nebuilder.neb.com/) (Additional file 1). We describe here the construction of the *hasS* deletion as an example, and provide sequencing confirmation for the *vreA* and *vreI* deletion constructs (Additional file 2). The flanking primers for the *hasS* deletion construct are as follows: upstream forward, 5′ cgggtaccgagctcgGCAAGACCCGCGATCCGC 3′ and upstream reverse, 5′ cgcgccgcgtgcggctCTCGTTGGTC 3′; downstream forward, 5′ agccgcacgcggcgcgATCGCTTCCG 3′ and downstream reverse, 5′ ctatgaccatgattacgGCAAGTATTCGCCGTGCACCG 3′ (overlapping bases in lower case, which facilitate fusion as shown in Fig. 1). The PCR reactions contained 200 μM dNTPs, 0.5 μM of forward and reverse primers, 10 ng/μl *P. aeruginosa* genomic DNA, 0.02 U/μl Q5 high fidelity DNA polymerase (New England Biolabs) in 1× reaction buffer containing 2 mM MgCl_2_ supplied by the manufacturer. The thermo cycles were programmed as follows: initial denaturation at 98 °C for 3 min, followed by 30 cycles of 98 °C for 10 s, annealing temperature for 30 s, and 72 °C for 15 s, and a final extension at 72 °C for 2 min. The PCR products were analyzed by DNA agarose electrophoresis and purified by QIAquick spin-column (QIAGEN) before use.

### Gibson assembly of the deletion construct

The pEX18Tc suicide plasmid was linearized within the multiple cloning site by restriction digest with *Eco*RI. The Gibson assembly reaction was performed as described by Gibson et al. [2]. The procedure is illustrated in Fig. 1. Briefly, the reaction mix contained 10 nM of each amplified upstream and downstream genomic DNA fragment, 5 nM of linearized pEX18Tc plasmid, 0.004 U/μl of T5 exonuclease, 4 U/μl of *Taq* ligase, 0.0125 U/μl of Q5 DNA polymerase, 5% (*W*/*V*) of PEG8000, 1 mM of NAD, 0.25 mM of dNTPs in 1× Q5 DNA polymerase reaction buffer containing 2 mM MgCl_2_. Individual reagents or master mix can be acquired from New England Biolabs. The reaction mixture was incubated at 50 °C for 1 h and the resulting ligated plasmid was transformed into high efficiency chemically competent *E. coli* DH5α (New England Biolabs). Transformants were checked by colony PCR using the universal pEX18 vector primers, which flank the multiple cloning site: forward, 5′ GGCTCGTATGTTGTGTGGAATTGTG 3′ and reverse, 5′ GGATGTGCTGCAAGGCGATTAAG 3′ (annealing temperature: 55 °C). Positive clones were further verified by DNA sequencing (Eurofins Scientific). A colony containing the sequenced deletion suicide vector was inoculated in TB medium with 10 μg/ml tetracycline and shaken at 250 rpm, 37 °C for about 23 h. Plasmid DNA was extracted by QIAprep spin miniprep kit (QIAGEN).

### Electroporation of *P. aeruginosa* and selection of the first homologous recombination

A variety of electroporation conditions [11–14] were tested and a method modified on the procedure described by Choi et al. [11] was used. One fresh colony of *P. aeruginosa* PAO1 was inoculated in 5 ml LB medium and grown overnight at 42 °C without shaking until reaching stationary phase. Cells from overnight cultures (4 ml) were harvested by centrifugation at 13000×g for 1 min at room temperature (~23 °C). The cell pellet was resuspended in 1 ml of 1 mM room temperature MgSO_4_ followed by centrifugation and the supernatant was discarded. The cells were washed again and the cell pellet was resuspended in 50 μl of 1 mM room temperature MgSO_4_. 5–10 μg of plasmid DNA was mixed with 50 μl of cell mixture, and the resulting mixture was transferred to a 2 mm gap electroporation cuvette (Sigma). Various pulsing parameters were tested for maximum transformation efficiency. Optimum electroporation conditions for PAO1 were determined to be 2.2 KV with a decaying exponential waveform (25 μF capacitor, 600 Ω) in a 2 mm gap cuvette at room temperature (see Results section Fig. 3). After pulsing on a Bio-Rad MicroPulser, 1 ml of BHI medium was added to the cuvette immediately and the mix incubated at room temperature for 5 min. The cells were transferred into a falcon tube and shaken at 37 °C for 3 h. Transformed cells were pelleted and 700 μl of the supernatant was discarded. The cells were resuspended in the remaining medium and plated on two to three BHI agar plates containing 100 μg/ml of tetracycline (BHI/Tc100). The plates were incubated at 37 °C for up to 64 h with recombinants generally appearing within the first 48 h.

### *sacB* counter-selection of the second homologous recombination

Colonies growing on the BHI/Tc100 plates were checked by colony PCR using the pEX18 universal forward primer and a primer specific to the genomic DNA following the cloned 500 bp downstream sequence (5′ ACATCTGCACCAGGTCGTC 3′), as illustrated in Fig. 4. The pEX18 universal reverse primer and a primer specific to the genomic DNA before the cloned 500 bp upstream sequence could also be used to screen recombinants. Positive colonies were purified by streaking on new BHI/Tc100 plates. Purified merodiploids (~ 2 mm) were streaked on TYS10 plates using a single loop/tip and incubated at room temperature (~ 23 °C). Colonies that lost the integrated plasmid usually appear within 48–60 h. Colonies were examined by colony PCR using a set of primers flanking the genomic sequence of the deleted locus *hasS* (forward, 5′ GATTACCGAGTCTTGCCGGTTC 3′, and reverse, 5′ ACATCTGCACCAGGTCGTC 3′) as illustrated in Fig. 4. Colonies with the confirmed deletion were purified by streaking on a TYS10 plate and further verified by DNA sequencing (Eurofins Scientific).

## Results

### Rapid and accurate construction of allelic exchange vectors using Gibson assembly

**Fig. 2:**
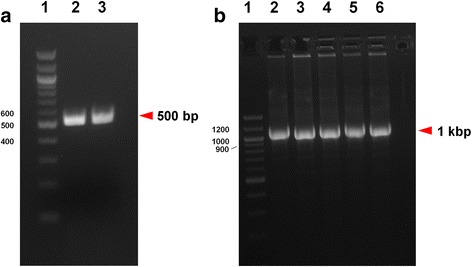
**a** Amplification of the 500 bp upstream and downstream DNA fragments of *hasS*. Lane 1, 400 ng DNA ladder; lane 2, 3 μl of the 500 bp PCR product of the upstream genomic DNA sequence of *hasS*; lane 3, 3 μl of the 500 bp PCR product of the downstream genomic DNA sequence of *hasS*. **b** Colony PCR of fused deletion alleles. The 500 bp upstream DNA fragment and the 500 bp downstream DNA fragment were fused and cloned into the pEX18Tc vector via Gibson assembly. Lane 1, 400 ng, 100 bp DNA ladder; lane 2–6, 10 μl of PCR products of five colonies randomly selected, which all showed the 1 kbp fused deletion alleles

The construction of the allelic exchange vector for gene *hasS* was performed as shown in Fig. 1. First, the 500 bp upstream and downstream DNA sequences flanking the locus of interest were amplified (Fig. 2a). Gibson assembly was used to fuse and clone these fragments into the pEX18Tc allelic exchange vector as described in the Materials and Methods. Other high fidelity DNA polymerase such as Phusion DNA Polymerase can also be used in the PCR and Gibson assembly. The resulting deletion construct was transformed into *E. coli* DH5α. Colonies containing the deletion construct were confirmed by colony PCR using the universal pEX18 primer pair flanking the multiple cloning sites (Fig. 2b). Positive colonies containing both the upstream and downstream DNA fragments showed a 1 kbp insert and were further selected for DNA preparation and sequencing. The accuracy of the Gibson assembly was extremely high with more than 95% of the colonies tested during method development (*n* = 28) containing the mutant deletion allele with the correct sequence.

The pEX18 series of suicide vectors have been widely used for site-directed gene manipulation in *P. aeruginosa* and related bacteria [7]. The construction of a gene knockout vector usually involves several steps of subcloning. To accelerate the procedure, Wolfgang et al. [15] and Choi et al. [11] used overlap PCR and the Gateway cloning technology (Invitrogen) to generate gene deletion constructs. However, the Gateway technology procedure still requires several extra cloning steps: first, the upstream and downstream fragments are amplified by flanking PCR; secondly, the two fragments are fused together by overlap PCR; and finally the resulting deletion allele is purified by electrophoresis and cloned into a Gateway allelic exchange vector. Furthermore, the pEX18 suicide vectors must be modified to be compatible with the Gateway cloning system by adding the *att* flanking sites. Gateway proprietary reagents such as Clonase are used to recombine the deletion allele from the Gateway vector into the modified Gateway compatible pEX18 vector. Utilizing the method described herein the complete construct can be generated in two steps: first, the upstream and downstream DNA fragments are amplified by PCR; then the DNA fragments are ligated directly into a linearized pEX18 plasmid by single step Gibson assembly to generate the allelic exchange vector. Neither proprietary reagents nor modifications to the suicide vector are required.

### Quick deletion construct transfer by electroporation and merodiploid selection

The transfer of the allelic exchange plasmid into *P. aeruginosa* is traditionally performed via conjugation. Specifically, the deletion construct is transformed into a conjugative *E. coli* strain, which is subsequently mated with recipient *P. aeruginosa* cells. After mating, transformed *P. aeruginosa* cells are isolated from the conjugation mixture by plating on media containing both triclosan and the antibiotic compatible with the plasmid resistance marker. Repetitive passage of the deletion construct under harsh double antibiotic selection places additional stress on the cells and may promote undesirable spontaneous mutations. Alternatively, we pursued a more direct approach via electroporation that avoids the multiple steps that may cause spontaneous mutations while shortening the protocol. Although transformation of *P. aeruginosa* by electroporation methods has previously been considered relatively inefficient, we have optimized the electroporation protocol described herein to transformation efficiencies similar to those obtained by conjugation.

To inhibit the DNA restriction-modification system of *P. aeruginosa*, the cells were grown at 42–43 °C overnight without shaking as described by Rolfe et al. [10]. To optimize the transformation efficiency, a panel of electroporation solutions was tested. Significant cell lysis was observed when water, 10–15% glycerol, HEPES buffer, 300–500 mM sucrose, 300–500 mM sorbitol or various combinations thereof were used. MgSO_4_ at 1 mM was found to effectively maintain cell structure integrity and not interfere with electroporation. Therefore, cells were harvested and washed in 1 mM MgSO_4_ at either 4 °C or 23 °C.

**Fig. 3:**
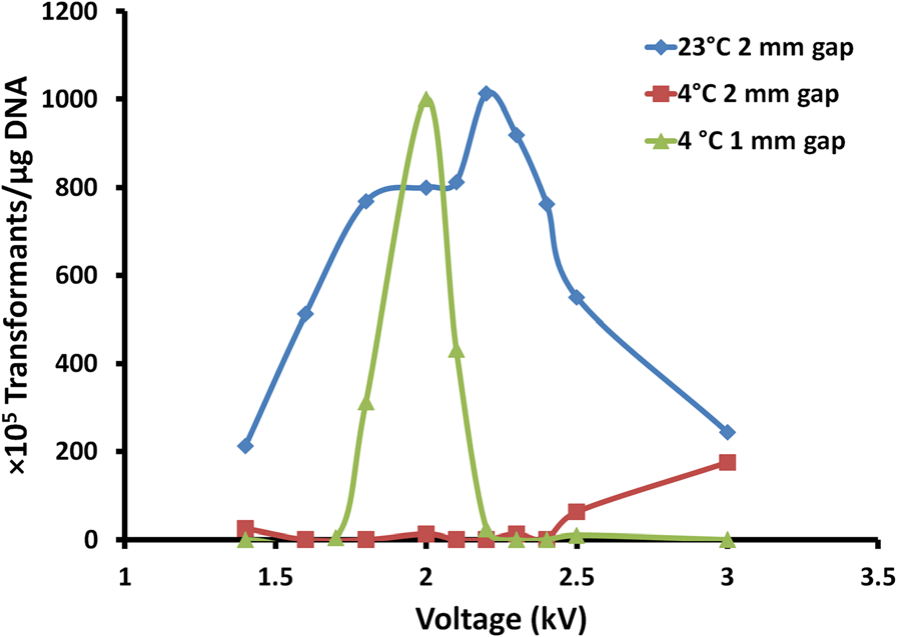
The transformation efficiency of *P. aeruginosa* PAO1 with pBSP11Tc^R^ under various electroporation conditions. The maximum efficiency was achieved at 2.0 kV using a 1 mm gap cuvette at 4 °C or at 2.2 kV using 2 mm cuvette at 23 °C. The field strength applied at 4 °C in a 1 mm gap cuvette is 20 kV/cm. Since the membrane permeation voltage is doubled at 4 °C as compared to room temperature [17], the field strength at 23 °C in a 2 mm cuvette (11 kV/cm) is equivalent to 22 kV/cm at 4 °C. Therefore, the optimum field strength is determined to be 20–22 kV/cm (4 °C equivalent). Since the 2 mm gap cuvette is less prone to arcing, electroporation at 2.2 kV using a 2 mm gap cuvette at room temperature was used to transform the suicide pEX18Tc allelic exchange construct into *P. aeruginosa* PAO1. Pulse durations of 4.0 to 4.5 ms were used in most experiments

The optimum electric parameters were investigated by testing the transformation efficiency of a *P. aeruginosa* replicative plasmid pBSP11Tc^R^ at both 4 °C and 23 °C using 1 mm gap and 2 mm gap cuvettes at different voltages. The efficiencies of electroporation under these conditions are shown in Fig. 3.

**Fig. 4:**
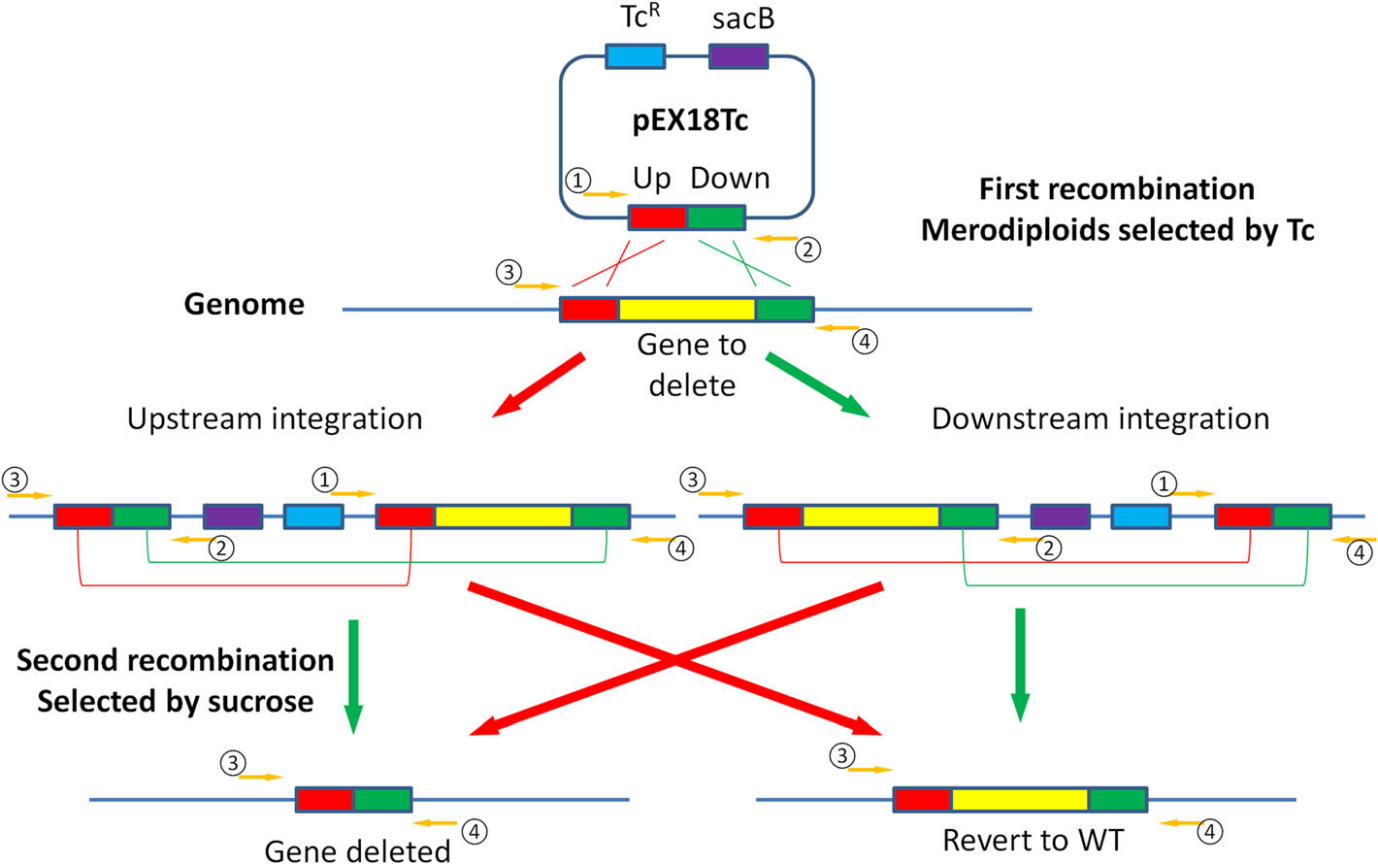
Gene knockout of *P. aeruginosa* via homology recombination. Primer 1 and 2, universal pEX18 forward and reverse primers flanking the multiple cloning sites, respectively. Primers 3 and 4, specific to genomic DNA sequences flanking the sequences cloned into the pEX18Tc vector. Primers 1 and 2 were used to screen *E. coli* DH5α transformants containing the deletion allele plasmid. Primers 1 and 4 or primers 2 and 3 were used to screen merodiploids in which the deletion construct was integrated into the chromosome. Primers 3 and 4 were used to screen deletion mutants following secondary recombination

Using these conditions the deletion construct was transferred into *P. aeruginosa* cells and integrated into the PAO1 chromosome by homologous recombination as shown in Fig. 4. Merodiploids in which the deletion construct is integrated into the chromosome usually appeared on the tetracycline selection plates within 40 h. In some cases, positive colonies appeared up to 60 h after electroporation. Beyond 72 h, spontaneous tetracycline resistant colonies began to appear. Suicide pEX18 vector constructs with alternate antibiotic resistance markers such as carbenicillin and gentamicin were also tested. However, colonies with spontaneous resistance against these antibiotics appeared as early as 20 h, which made selection of merodiploids more difficult. Therefore, tetracycline resistance appears to provide the most optimal selection marker for merodiploids by this method.

**Fig. 5:**
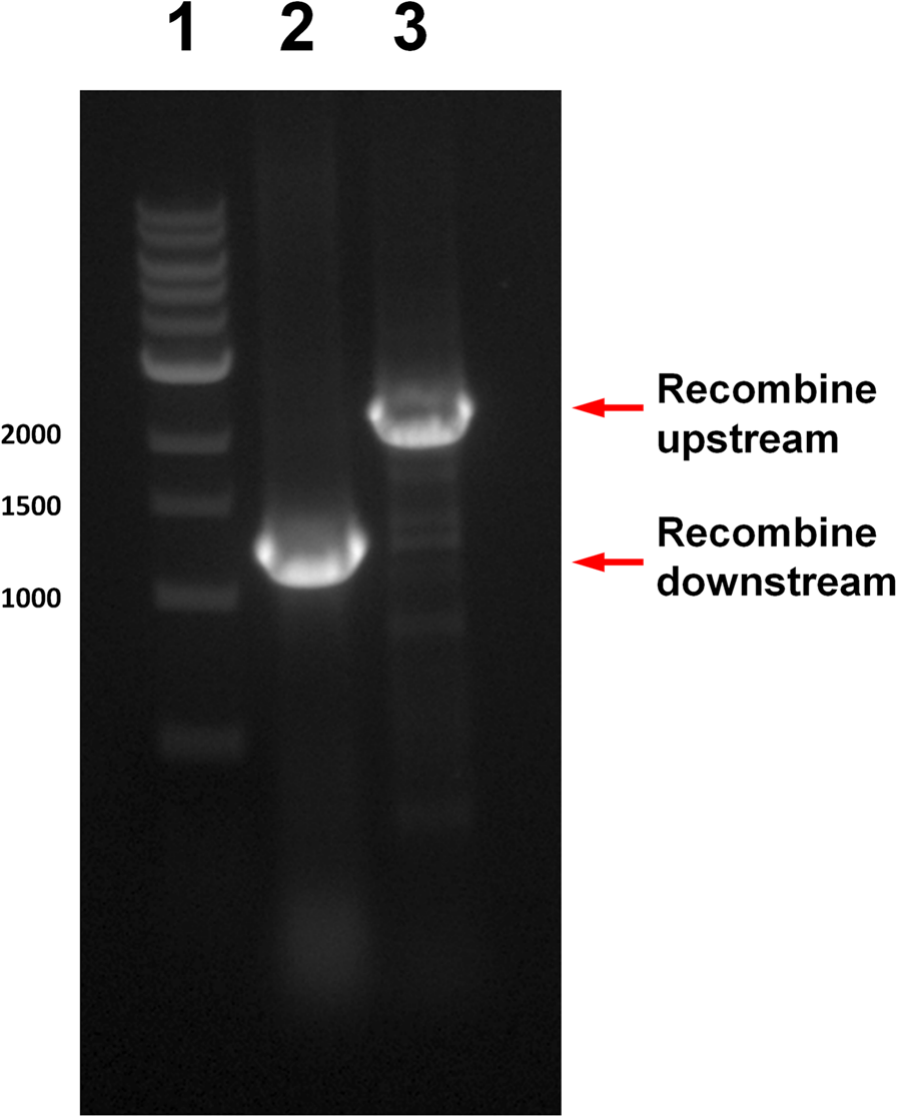
Colony PCR of merodiploids. Lane 1, 400 ng 1 kB DNA ladder; lane 2, 10 μl of PCR product of a colony in which plasmid integrated via the downstream homologous sequence; lane 3, 10 μl of PCR product of a colony in which plasmid integrated via the upstream homologous sequence

Tetracycline resistant colonies were analyzed by colony PCR to confirm the generation of merodiploids (Fig. 5). Using the universal pEX18 vector forward primer (primer 1) and a primer specific to a genomic sequence after the 500 bp downstream sequence (primer 4), colonies in which the plasmid integrated via the upstream homologous sequence yielded a ~ 2 kbp PCR product (Fig. 5, lane 3). Meanwhile, colonies in which the plasmid integrated via the downstream homologous sequence yielded a ~ 1 kbp band (Fig. 5, lane 2). The approximate crossovers for either of these crossover events are illustrated in Fig. 4. If the PCR was performed with the universal pEX18 vector reverse primer (primer 2) and a primer specific to a genomic sequence before the 500 bp upstream (primer 3), reversed patterns would be expected. Generally, 5–10 tetracycline resistant colonies were obtained, all of which were confirmed to be merodiploids by PCR.

### Improved sucrose-*sacB* counter-selection of knockout mutants

Following confirmation by colony PCR, merodiploids were isolated by streaking selected colonies onto a fresh tetracycline selection plate to avoid carryover contamination. Isolated colonies were then streaked onto TYS10 plates containing 300 mM sucrose to select for colonies that have lost the integrated plasmid backbone containing *sacB*. The *sacB* gene was originally isolated from *Bacillus subtilis* and encodes levansucrase, which catalyzes the conversion of sucrose to levans, a molecule that is toxic to *P. aeruginosa*. The sucrose intolerance of merodiploids expressing *sacB* is caused by accumulation of levans in the periplasmic space, which is a slow process and does not cause immediate lethality. Direct streaking of tetracycline resistant colonies onto TYS10 plates and the alternative streaking from an intermediate culture in LB were directly compared and no difference was observed. However, considering the possibility that a wild type revertant may gain a growth advantage in an intermediate culture and outcompete the deletion mutant, streaking an isolated tetracycline resistant colony directly onto a TYS10 plate minimizes undesirable population bias arising prior to counter-selection. The TYS10 medium contains no salt and thus increases the sensitivity of cells to the accumulation of levans [16]. The *sacB* counter-selection was performed at room temperature to reduce sucrose hydrolysis and slow down the growth of *P. aeruginosa* to allow more effective selection of merodiploids having undergone the second homologous recombination event.

**Fig. 6:**
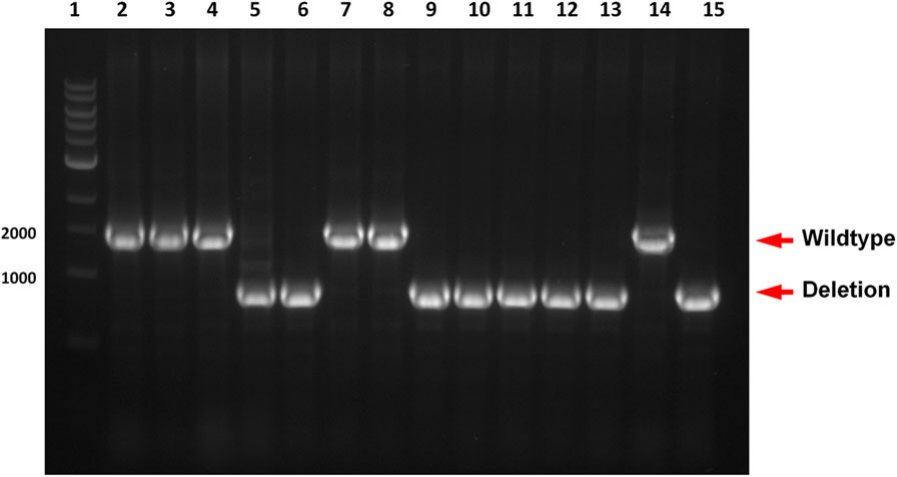
Screen of deletion mutants by colony PCR. Lane 1, 400 ng 1 kb DNA ladder; lane 2–15, 10 μl of PCR products of 14 colonies randomly selected from the TYS10 plate. Of the fourteen randomly selected colonies, 8 colonies were deletion mutants while six colonies reverted to wild type during the second recombination event

Sucrose-resistant colonies were analyzed by colony PCR. As illustrated in Fig. 4, two populations formed: one included the desired deletion mutants while the other included wild type revertants. The theoretical ratio between two populations is 1:1 and in the case of *hasS*, the ratio of Δ*hasS* vs wild type of 14 randomly selected colonies was 8:6, as judged by the 2 kb versus 1 kb insert, respectively (Fig. 6). However, the ratio may be biased towards one population if for example the gene to be deleted is beneficial for bacterial growth. The deletion of *hasS* was confirmed by DNA sequencing (Additional file 3).

## Discussion

In this study, we present a rapid and efficient method to create unmarked genetic knockouts of *P. aeruginosa*. In comparison to the previous two-step allelic exchange procedure, which usually takes a minimum of two weeks, this protocol can be completed in one week. Even when compared to the Gateway recombination method described by Choi et al. [9], the time required in the current protocol is reduced and does not depend on proprietary reagents and reengineering of the suicide vector to make it Gateway compatible.

The traditional procedure requires several extra sub-cloning steps to create the deletion construct, which is complicated by dependence on available restriction sites and the potential for low DNA recovery from agarose gels. In our method, the suicide vector can be simply linearized by any restriction site within the multiple cloning site. The amplified genomic DNA fragments can then be assembled with the linearized vector in one step by Gibson assembly, which is straightforward to set up and does not require any expensive proprietary reagents or instruments. The accuracy of Gibson assembly combined with the desirable construct being obtained with 95% efficiency (*n* = 28) makes this a viable and attractive alternative to previous methods.

In the engineered gene deletion constructs, the amplified genomic DNA sequence is fused exactly as designed without introducing any other changes to the sequence. The seamless fusion allows for in-frame deletion and leaves no residual nucleotides behind, minimizing polar effects, which is especially important when studying polycistronic operons. The precision of the method is exemplified in the *vreA* and *vreI* constructs where the stop codon of *vreA* and the start codon of *vreI* overlap. The current method allows for the generation of the *vreA* and *vreI* deletion mutants to single base pair precision while maintaining the start and stop codon of the respective genes (see Additional file 2).

The transformation of the mutant allele construct into *P. aeruginosa* by electroporation provides a fast and straightforward alternative to conjugation. Repetitive microbiological procedures are minimized, reducing workload while greatly reducing the time to complete the two-step allelic exchange. Moreover, direct deletion allele transfer without having to passage *P. aeruginosa* repetitively, as required following conjugation, may reduce the opportunity for introduction of suppressor mutations.

Besides construction of *P. aeruginosa* deletion mutants, this method can also be used to create insertion mutants and locus replacement mutants. Moreover, the protocol may be readily adapted for other related organisms. The fast and streamlined procedure also allows for construction of a panel of mutants in a reasonable timeframe, which can greatly increase the throughput of reverse genetics research.

## Conclusion

*Pseudomonas aeruginosa* represents a model organism for biological study of biofilm formation and quorum sensing. However, genetic manipulation in *P. aeruginosa* traditionally requires multiple cloning steps in addition to several selective passages following conjugation. Herein we describe a rapid and efficient means of generating deletion mutants using Gibson assembly and direct transformation removing several steps of DNA manipulation and passaging, reducing the likelihood of introducing mutations and without the need for proprietary reagents.

## Additional files

Additional file 1: Oligonucleotide primers used in this study. (DOCX 71 kb)

Additional file 2: Sequencing results for the vreA (A) and vreI (B) deletion strains. (DOCX 10162 kb)

Additional file 3: Sequencing results for the hasS deletion mutant. (DOCX 5552 kb)

## Abbreviations

BHI: Bovine heart infused media
ECF: Extra cytoplasmic function
has: Heme assimilation system
LB: Luria broth
PCR: Polymerase chain reaction
TB: Terrific broth
TYS10: Tryptone, yeast extract, sucrose broteh
vre: Virulence regulator involved ECF system
