# Supplementary material for: A rapid seamless method for gene knockout in Pseudomonas aeruginosa
Source: BMC Microbiol. 2017 Sep 19;17:199. doi: 10.1186/s12866-017-1112-5 (PMC5606073; doi:10.1186/s12866-017-1112-5)
Supplement: Supplementary file 1 — Oligonucleotide primers used in this study. (DOCX 71 kb) [file 12866_2017_1112_MOESM1_ESM.docx]

| Primer name | Primer sequence |
| --- | --- |
| *hasS* upstream forward | cgggtaccgagctcgGCAAGACCCGCGATCCGC |
| *hasS* upstream reverse | cgcgccgcgtgcggctCTCGTTGGTC |
| *hasS* downstream forward | agccgcacgcggcgcgATCGCTTCCG |
| *hasS* downstream reverse | ctatgaccatgattacgGCAAGTATTCGCCGTGCACCG |
| *vreA*  upstream forward | cgggtaccgagctcgGACCTGCGGAAGCCCTTA |
| *vreA* upstream reverse | ccctccgtCATCAACGGATTCCAGTCC |
| *vreA* downstream forward | cgttgatgACGGAGGGAGTGGGAGGG |
| *vreA* downstream reverse | ctatgaccatgattacgCTTTTCTTTCCAGGCGCTCCG |
| *vreI*  upstream forward | cgggtaccgagctcgGGCCCGTGGACGGCGCTC |
| *vreI* upstream reverse | cttatgcttaGCTCATGACCCTCCCACTCCCTC |
| *vreI* downstream forward | tcatgagcTAAGCATAAGCAGGTGGAAAACCGC |
| *vreI* downstream reverse | ctatgaccatgattacgCACGTTCAGGCGGGTGCG |

Additional file 1. Oligonucleotide primers used in this study.
