# Supplementary figures and images for: A rapid seamless method for gene knockout in Pseudomonas aeruginosa
Source: BMC Microbiol. 2017 Sep 19;17:199. doi: 10.1186/s12866-017-1112-5 (PMC5606073; doi:10.1186/s12866-017-1112-5)

A.


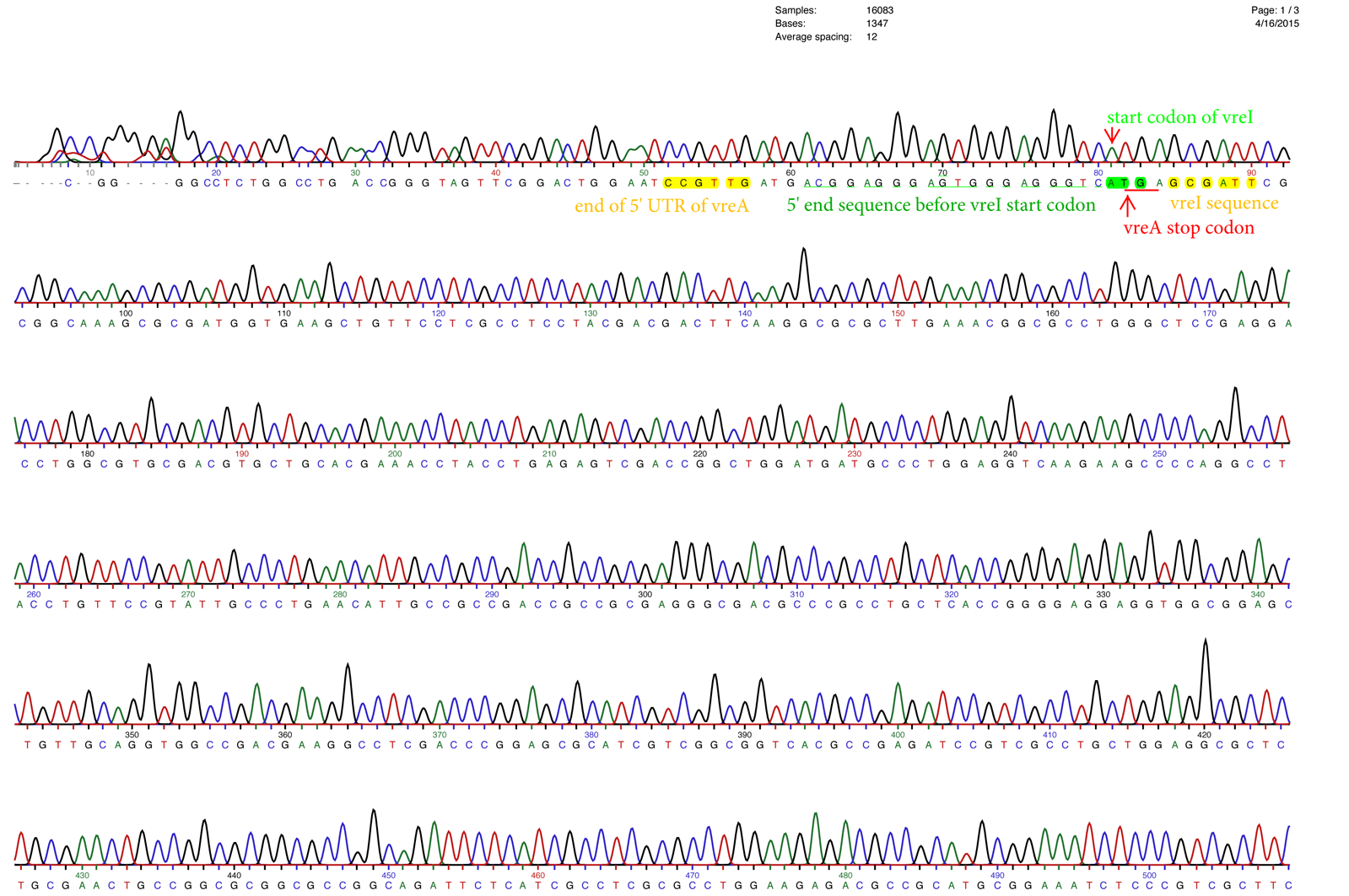


B.


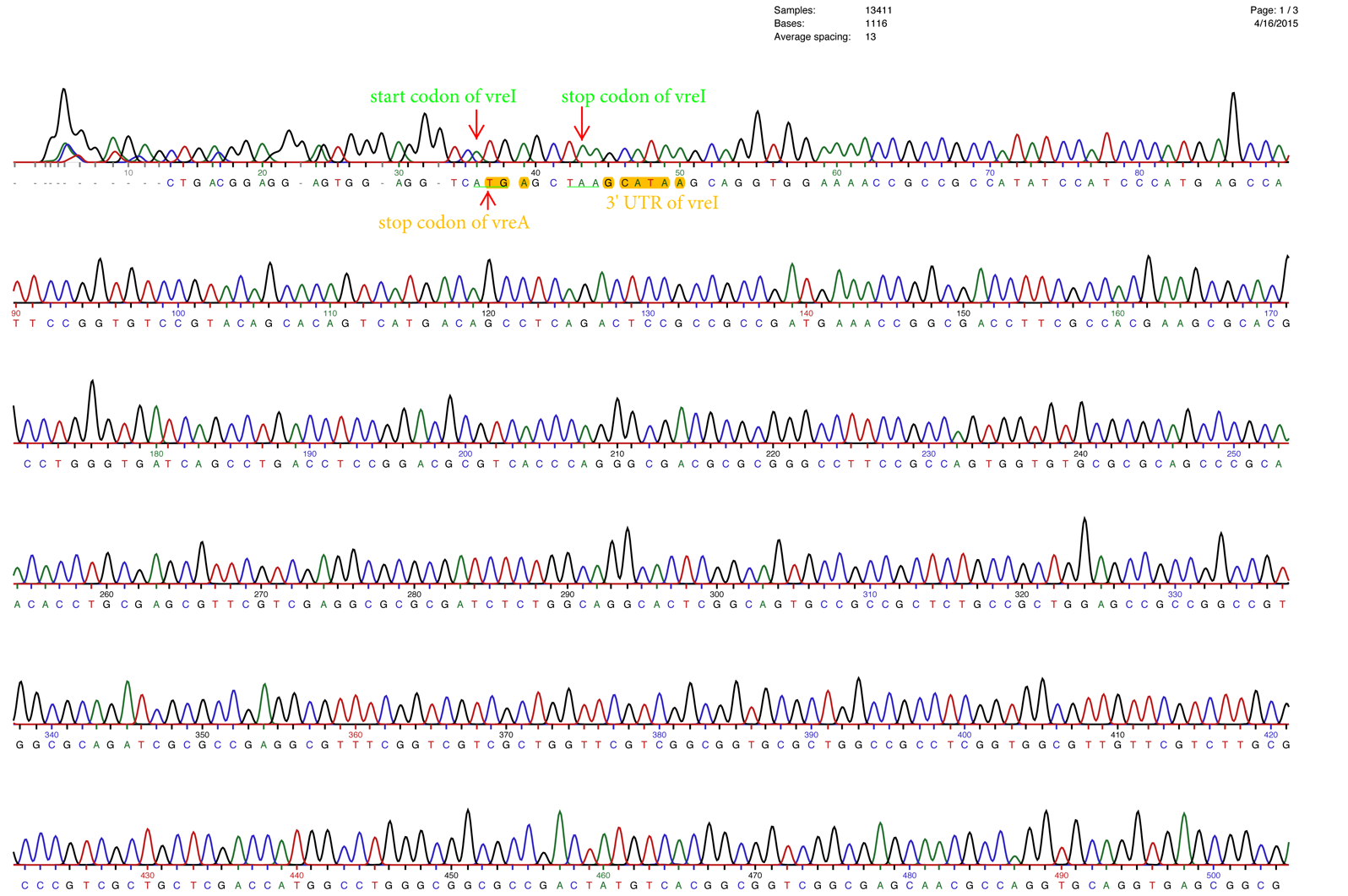


Additional file 2. Sequencing results for the *vreA* (A) and *vreI* (B) deletion strains.

Supplement: Supplementary file 2 — Sequencing results for the vreA (A) and vreI (B) deletion strains. (DOCX 10162 kb) [file 12866_2017_1112_MOESM2_ESM.docx]

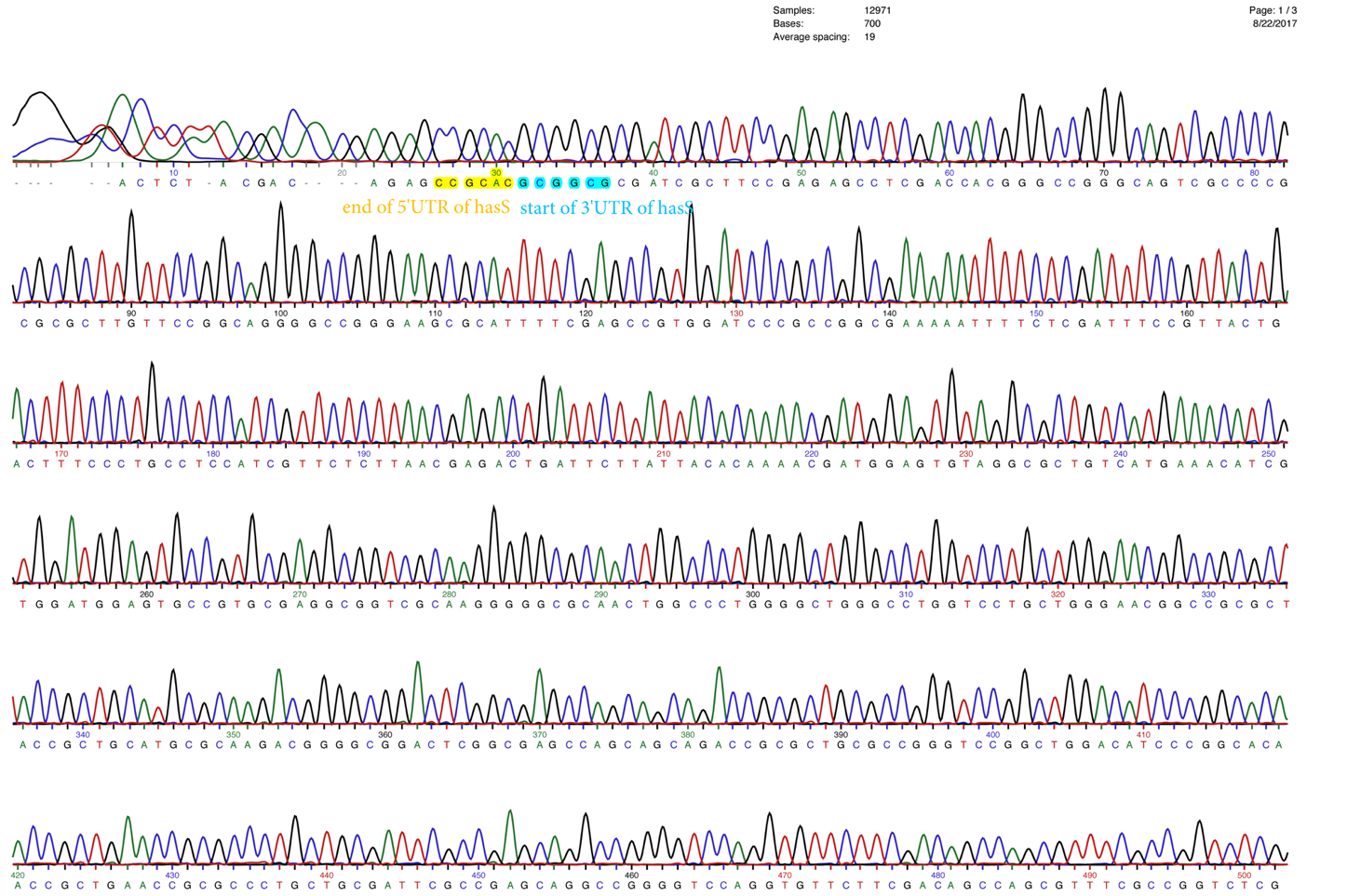


Additional file 3. Sequencing results for the *hasS* deletion mutant.

Supplement: Supplementary file 3 — Sequencing results for the hasS deletion mutant. (DOCX 5552 kb) [file 12866_2017_1112_MOESM3_ESM.docx]
